# Optimization of Flavonoid Extraction from *Abelmoschus manihot* Flowers Using Ultrasonic Techniques: Predictive Modeling through Response Surface Methodology and Deep Neural Network and Biological Activity Assessment

**DOI:** 10.3390/molecules29112610

**Published:** 2024-06-01

**Authors:** Zenghong Sun, Wenhui Liu, Sha Zhang, Shuge Tian, Ainiwaer Aikemu

**Affiliations:** 1 College of Traditional Chinese Medicine, Xinjiang Medical University, Urumqi 830017, China; sunzenghong@stu.xjmu.edu.cn (Z.S.); zhangsha@stu.xjmu.edu.cn (S.Z.); 2 College of Information Sciences and Technology, Xinjiang Education Institute, Urumqi 830043, China; liuwhks@163.com; 3 Xinjiang Key Labaratory of Hetian Characteristic Chinese Traditional Medicine Research, Hetian 843000, China

**Keywords:** *Abelmoschus manihot* flower, ultrasonic cell crusher, green extraction, dental caries, *Streptococcus mutans* (*S. mutans*), antioxidant activity

## Abstract

Understanding the optimal extraction methods for flavonoids from *Abelmoschus manihot* flowers (AMF) is crucial for unlocking their potential benefits. This study aimed to optimize the efficiency of flavonoid extraction from AMF. After comparing extraction methods, the ultrasonic cell crusher demonstrated superior performance over conventional techniques. Four key factors—solid-to-liquid ratio (1:10 to 1:50 g·mL^−1^), ethanol concentration (55% to 95%), ultrasonic time (10 to 50 min), and ultrasonic power (5% to 25% of 900 W)—were investigated and normalized using the entropy weight method. This led to a comprehensive evaluation (CE). Optimization of extraction conditions for the ultrasonic cell crusher was achieved through response surface methodology and a deep neural network model, resulting in optimal parameters: ethanol volume fraction of 66%, solid-to-liquid ratio of 1:21 g/mL, extraction efficiency of 9%, and extraction duration of 35 min, yielding a CE value of 23.14 (RSD < 1%). Additionally, the inhibitory effects of the optimized extracts against *Streptococcus mutans* (*S. mutans*) were assessed. The results revealed that AMF extract (AMFE) exhibits inhibitory effects on *S. mutans*, with concomitant inhibition of sucrase and lactate dehydrogenase (LDH). The MIC of AMFE against planktonic *S*. *mutans* is 3 mg/mL, with an MBC of 6 mg/mL. Within the concentration range of 1/8 MIC to 2 MIC of AMFE, the activities of sucrase and LDH decreased by 318.934 U/mg prot and 61.844 U/mg prot, respectively. The antioxidant activity of AMFE was assessed using the potassium ferricyanide reduction and phosphomolybdenum methods. Additionally, the effect of AMFE on DPPH, ABTS, and ·OH free radical scavenging abilities was determined. The concentrations at which AMFE exhibited over 90% scavenging rate for ABTS and DPPH free radicals were found to be 0.125 mg/mL and 2 mg/mL, respectively.

## 1. Introduction

The dried flower of *Abelmoschus manihot* (AMF) is obtained from a plant belonging to the mallow family, specifically *Abelmoschus manihot* (L.) [1]. With a rich history of utilization in China, AMF has been widely used as a folk medicine in the treatment of chronic kidney disease and diabetes nephropathy in China, playing a significant role by reducing the content of urinary protein and protecting renal function [2,3]. Modern pharmacological research results indicate that AMF has multiple biological characteristics, including anti-inflammatory, antioxidant, anticonvulsant, and anti-tumor properties [4,5,6,7]. The primary constituents of AMF include flavonoids, predominantly hyperin (Hyp), isoquercitrin (Iso), rutin, and quercetin (Que) [1,8]. Within the fields of cosmetics, pharmaceuticals, and health-related dietary products, AMF plays a crucial role as a primary raw material or intermediate substance [9,10].

In recent years, the concept of “green extraction” of natural products has gained prominence in the public domain [11]. Its foundational principle entails the redesign of extraction procedures to diminish energy consumption, while concurrently ensuring the quality and safety of the extracts [12]. Ultrasonic extraction technology, a recently developed approach, has garnered extensive attention and research optimization owing to its broad applicability and cost-effectiveness [13]. 

The response surface model (RSM) deploys mathematical models to delineate the relationship between the input variables and resultant responses, consequently enhancing the extraction efficiency through experimental design and result analysis. RSM further provides insight and prognostication regarding experimental outcomes, affording researchers a comprehensive understanding of the impact of each parameter on the yield of the target compounds [14]. This methodology has widespread applications in process optimization across diverse domains. Furthermore, expanding on the utilization of artificial neural networks, it is worth noting that their application in the domain of bioactive compound extraction has shown significant promise in recent years. Deep neural networks (DNNs), characterized by their multiple layers and complex architecture, have emerged as the preferred choice for modeling intricate relationships within datasets. By emulating the interconnected structure of neurons in the human brain, DNNs are capable of processing vast amounts of data and extracting meaningful patterns that can aid in the optimization of extraction processes [15]. In this research endeavor, the synergistic combination of response surface methodology and DNN models was utilized to determine the optimal process parameters.

The human oral cavity, gastrointestinal tract, and integumentary system are crucial ecological niches that are susceptible to colonization by commensal microorganisms. Research has elucidated the substantive association between gut bacteria and several human diseases, including inflammatory bowel disease, type 2 diabetes, colorectal cancer, and cardiometabolic disorders [16,17,18,19,20]. While certain pathogenic bacteria cause diseases, prevalent gut bacterial species can also contribute to disease onset or progression [21]. Notably, the oral microbiome, second only to the gut in terms of microbial abundance and diversity, harbors specific bacterial strains linked to oral pathologies, notably dental caries [22]. *S. mutans*, recognized widely as a cariogenic pathogen, is noteworthy for its ability to form biofilms and release virulence factors [23,24]. Lactate dehydrogenase (LDH) in *S. mutans* produces acid through carbohydrate metabolism and accumulates in the plaque biofilm, causing local acidity in the dental plaque to be too high, in turn causing mineralization and detachment of enamel leading to the occurrence of dental caries [25]. *S. mutans* use carbohydrates such as sucrose to synthesize organic acids and induce caries. The activity of sucrase reflects the ability of cariogenic bacteria to produce acid using sucrose, while the ability of cariogenic bacteria to synthesize sucrase reflects their cariogenic ability [26,27]. It can be concluded that inhibiting the activities of LDH and sucrase is of great significance in reducing the occurrence of dental caries. Conventional therapeutic approaches involving fluoride and antibiotics, while effective to an extent, are burdened by the long-term consequences of bacterial resistance to antibiotics and associated toxic side effects [28]. In recent years, researchers have directed their attention towards utilizing natural products for promoting oral health and preventing dental caries [29]. Natural products, known for their broad spectrum of pharmacological effects and excellent biocompatibility, serve as a crucial source of oral antimicrobial agents, including flavonoids [30,31,32]. Free radicals, chemical entities characterized by unpaired electrons, are produced within the body and play diverse roles in biochemical processes. While these radicals are essential for physiological functions, an imbalance caused by excessive or insufficient scavenging can have detrimental effects on human health. Antioxidants found in natural products, including flavonoids, help mitigate damage to human cells and tissues induced by free radicals through neutralization and other mechanisms [31,33]. Given the antimicrobial and antioxidant properties exhibited by flavonoids in various plants, and the high content of flavonoid compounds in AMF, it is reasonable to infer that AMFE may possess antimicrobial activity against *Streptococcus mutans* and antioxidant capacity [34].

This study aimed to ascertain the optimal extraction conditions by optimizing five key components: total flavonoid compounds (TFC), rutin, Hyp, Iso, and Que, as well as the composite index CE of AMF. Moreover, we explored the antimicrobial and antioxidant effects under the auspices of optimal extraction parameters.

## 2. Results and Discussion

### 2.1. Comparison with Traditional Methods

In this study, the TFC, rutin, Hyp, Iso, Que content, and CE values of the composite indices were used as the main determinants, and the extraction processes of maceration, heated reflux, and ultrasonic cell crusher extraction methods were compared.

**Figure 1:**
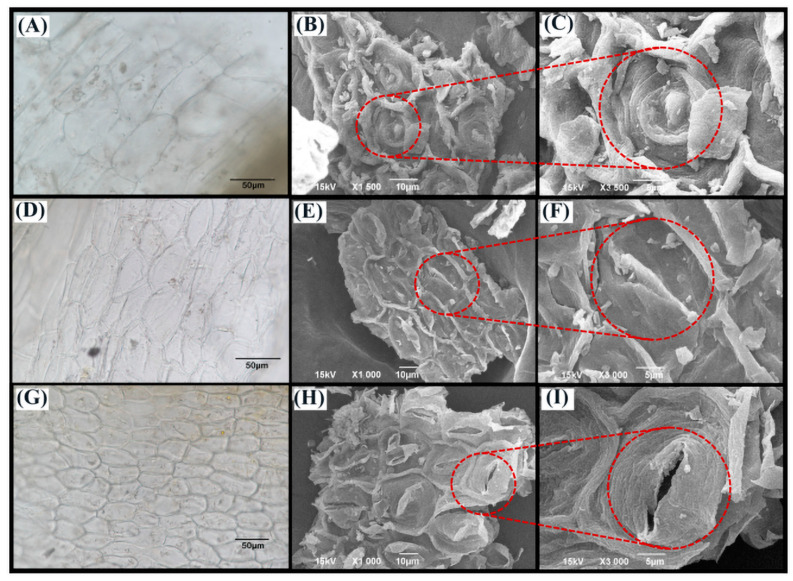
The OM and SEM images of AMF powder. (**A**–**C**) Maceration extraction, (**D**–**F**) reflux extraction, (**G**–**I**) ultrasonic extraction.

As shown in Table 1, the ultrasonic cell crusher extraction method exhibited superior performance compared to the other two traditional methods for extracting flavonoid components. Microscopy and scanning electron microscopy comparison diagrams were employed to further scrutinize the three extraction methods. Figure 1 shows conspicuous and denser cracks on the surface of plant cells subjected to the ultrasonic cell crusher extraction method. Additionally, the surface exhibited flocculent material. Conversely, no evident cracks were discernible in the images of the other two groups. In summary, it can be deduced that the ultrasonic cell crusher extraction method is more effective in disrupting plant cell structures and, consequently, in releasing target intracellular components more efficiently.

The maceration extraction method, which is conducted at room temperature, typically requires a longer extraction duration because of its slower pace. In contrast, heated reflux extraction relies on elevated solvent temperatures to expedite the penetration of solvent molecules through plant material pores and cell walls, facilitating the dissolution of solutes within the solvent. Consequently, compared with the maceration extraction method, heated reflux extraction accelerates the dissolution of the target components, leading to a reduction in the extraction time. However, for certain insoluble substances or compounds that exhibit strong structural stability, the effectiveness of the reflux extraction method may be compromised. In contrast, the ultrasonic cell crusher extraction method demonstrated a superior performance. This superiority can be attributed to the action of ultrasonic waves, which generate high-frequency vibrations that result in intense liquid shear and shock forces. These forces disrupt the plant cell wall, thereby releasing the intracellular target components. 

### 2.2. Single Factor Results

The weight values of TFC, rutin, Hyp, Iso, and Que were calculated sequentially by applying EWM based on the data of each evaluation index of the single factor, and the weight values of TFC, rutin, Hyp, Iso, and Que were 0.2931, 0.1436, 0.2712, 0.2563, and 0.0356, respectively. The CE of the comprehensive evaluation index was calculated after assigning weights = TFC × 0.2931 + rutin × 0.1436 + Hyp × 0.2712 + Iso × 0.2563 + Que × 0.0356.

**Figure 2:**
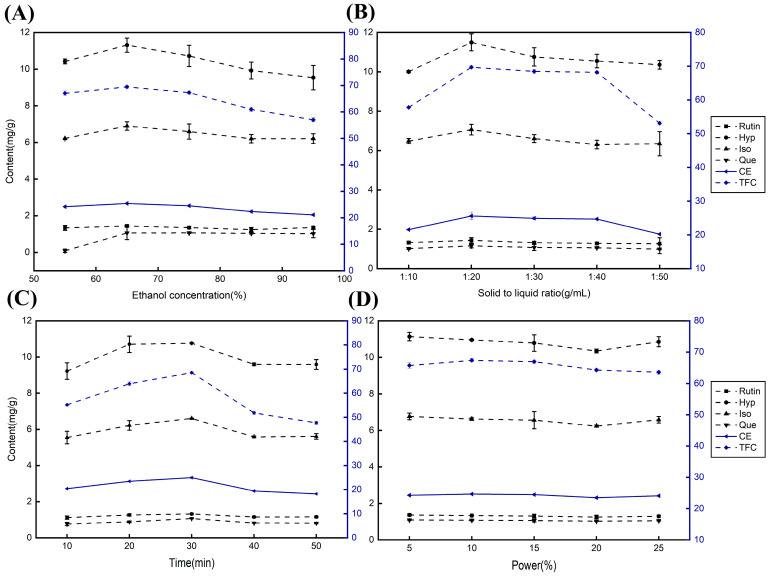
Effect of various factors on the content of active components from AMF. (**A**) ethanol concentration, (**B**) solid-to-liquid ratio, (**C**) extraction time, (**D**) ultrasonic power.

The influence of the ethanol volume fraction on the extraction yield and target ingredient content was assessed based on the results of a one-way experiment. Figure 2A illustrates that TFC, rutin, Hyp, Iso, Que, and the composite index CE all exhibit an upward trend with increasing ethanol volume fraction. The maximum extract yield was obtained when the ethanol concentration was 65%. Therefore, in the subsequent optimization experiments, an ethanol volume fraction of 65% served as an intermediate reference value for the design. This phenomenon can be attributed to the similar polarity of 65% ethanol to the target components in the sample, promoting a higher concentration gradient between the target components within the sample cells and solvent. Consequently, the leaching efficiency of the target components in the solvent was enhanced. Furthermore, filtration difficulties were encountered at an ethanol volume fraction of 50%, likely due to the challenge of dissolving mucilage in the herbs in a low ethanol concentration environment, rendering subsequent filtration problematic and unfavorable for target component extraction.

The selection of the solid-to-liquid ratio is a pivotal parameter affecting the extraction volume and is crucial for industrialized production. Figure 2B reveals that the contents of all target components and CE increased as the solid-to-liquid ratio increased, reaching their zenith at a solid-to-liquid ratio of 1:20 g/mL, and then exhibited a declining trend. In particular, TFC and CE showed steeper declines at solid-to-liquid ratios of 1:40 to 1:50 g/mL. Consequently, the subsequent optimization employed a solid-to-liquid ratio of 1:20 (g/mL) as an intermediate reference value for design purposes. The rapid leaching of active ingredients at lower solid-to-liquid ratios may lead to higher mass concentrations and saturation, inhibiting further dissolution. As the material–liquid ratio increased, the concentration gradient between the internal and external environments of the herb was amplified, facilitating the leaching of active ingredients. Once the solid-to-liquid ratio reached 1:20 (g/mL), most of the active ingredients likely reached saturation. Further increases in the material–liquid ratio would result in solvent waste and promote the leaching of other impurities.

Regarding the sonication time, the results depicted in Figure 2C exhibit analogous trends for TFC, rutin, Hyp, Iso, Que content, and CE. The extraction rates displayed an increasing trend within the 10 to 30 min range, peaking at 30 min and subsequently decreasing between 30 and 50 min. Consequently, 30 min was utilized as an intermediate reference value for the design in the subsequent optimization. This result can be attributed to the extraction of active ingredients from AMF, which reached completion at the 30 min mark. Prolonged ultrasonication leads to an increase in solution temperature, potentially inducing oxidative polymerization of flavonoids at high temperatures, which may compromise the structural integrity of flavonoids and reduce the content of the detected active ingredients.

Finally, in terms of ultrasonic power (Figure 2D), TFC, rutin, and Que content, along with CE, reached their peak values at 10%, whereas Hyp and Iso exhibited their highest values at 5%. In subsequent optimization experiments, 10% served as an intermediate reference value to facilitate determination of the optimum level. The ultrasonic cell crusher extraction method achieves optimal extraction efficiency with minimal ultrasonic power by disrupting the plant cell wall and liberating the target intracellular components through ultrasonic wave action [35].

### 2.3. Results of RSM

The experimental parameters and results of the 29 extraction conditions are listed in Table 2. A second-order polynomial equation regression fit was employed using Design Expert 12.0 to model these data, yielding a quadratic equation describing the relationship between the CE indicator and the respective variables. The Equation (1) elucidates how the responses vary as a function of extraction parameters.

(1) $$\begin{array}{c} {CE}_{pred}=23.07+0.7345A+0.7967B+0.0022C+0.6027D-0.1570AB-\\ 0.2065AC-0.0248AD-0.3537BC+0.1199BD+0.0890CD-0.7014A^{2}-\\ 1.92B^{2}-{1.26C}^{2}-1.26D^{2} \end{array}$$

The ANOVA results for the model are presented in Table 3. The model displayed a significant *p* value of less than 0.0001, and a non-significant *p* value (0.4299) of Lack of Fit greater than 0.05, indicating that it is adequately accurate and statistically significant, and thus capable of predicting correlated responses. Notably, while the correlation coefficient R^2^ was substantial (0.9241), it did not entirely capture the excellent fit of the regression model. Consequently, an adjusted R^2^ (R^2^_Adj_) was considered, which better reflected the representational capability of the model. In this experiment, the predicted and adjusted R^2^ values were 0.7418 and 0.8883, respectively, with the difference between them within reasonable limits, demonstrating consistency.

The coefficient of variation (C.V.) was used to compare the experimental data, with a lower C.V. value indicating higher precision and reliability. In this experiment, the C.V. was 2.25%, which was less than 10%, indicating highly precise and reliable experimental values. Furthermore, the Adequate Precision measurement of the Signal-to-Noise Ratio (Adeq Precision) was examined. An Adeq Precision value greater than 4 was considered desirable. In this model, the Adeq Precision value was 13.1487, indicating adequate signal. Thus, it can be concluded that the developed model is suitable for navigational design space applications.

Additionally, the ANOVA results of the model revealed that the effects of ethanol volume fraction, solid-to-liquid ratio, and extraction time on CE were highly significant (*p* < 0.001). Specifically, the primary terms A, B, and D, as well as the secondary terms A^2^, B^2^, C^2^, and D^2^, exert a significant influence on CE. Conversely, the primary term C and the cross terms AB, AC, AD, BC, BD, and CD had no significant impact on CE. Moreover, based on the regression equation of the model (Equation (1)), it can be observed that the magnitude of the positive impact of several single factors on CE follows the order of solid-to-liquid ratio (B) > ethanol volume fraction (A) > extraction time (D). Among the interaction factors, only BD and CD exhibit positive effects, with impact coefficients of 0.1199 and 0.0890 each.

**Figure 3:**
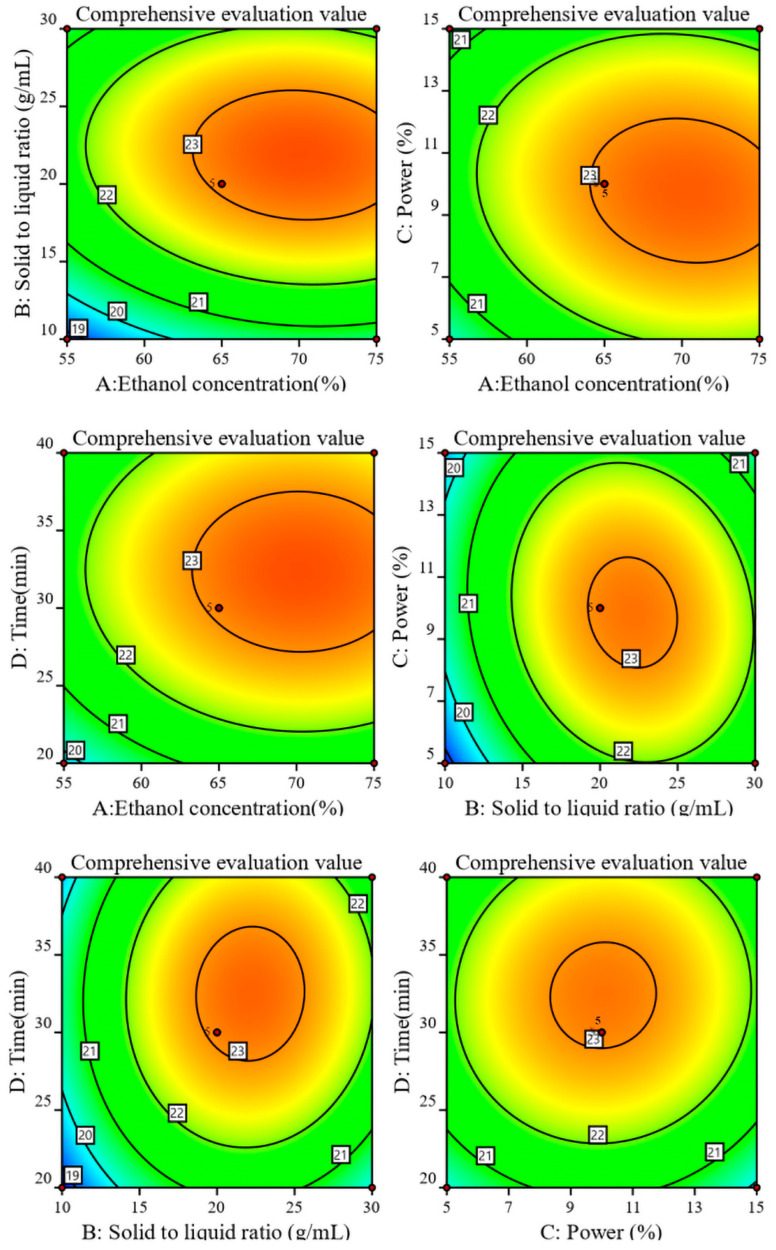
Contours of the influence of different factor interactions on CE. Note: The closer the color in the figure is to red, the higher the CE value, with the dots being the maximum value.

**Figure 4:**
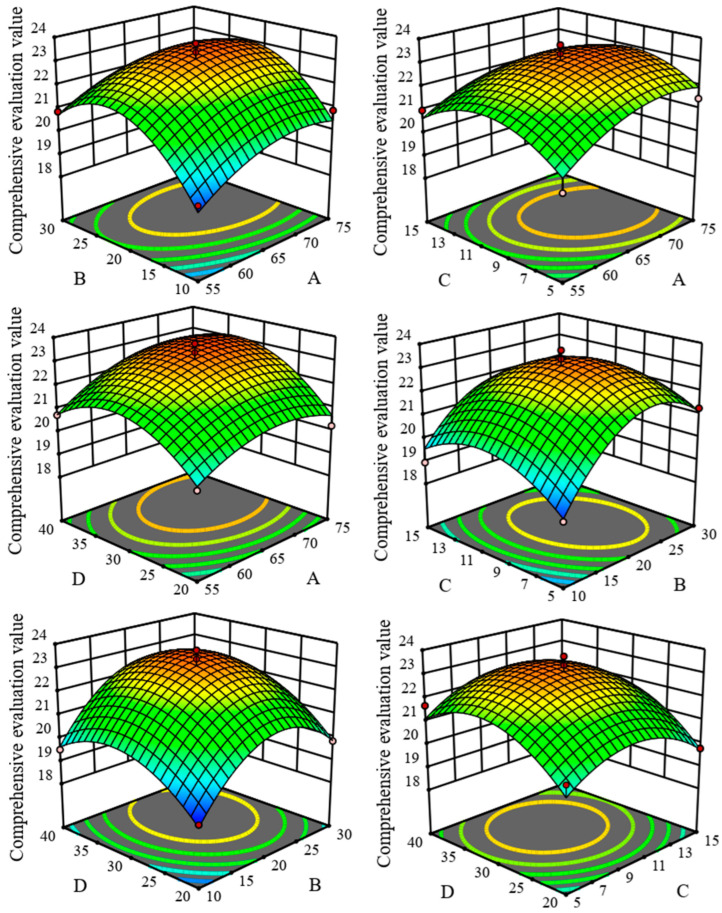
Three-dimensional response surface diagrams of the influence of different factor interactions on CE. A: ethanol concentration (%), B: liquid-to-solid ratio (g/mL), C: power (%), D: time. Note: The closer the color in the figure is to red, the higher the CE value, with the dots being the maximum value.

Figure 3 and Figure 4 depict the two-dimensional (2D) response surface plots and three-dimensional (3D) contour plots of the RSM model parameters generated using the second-order quadratic polynomial regression equation. These graphical representations offer visual insights into the relationships between the experimental factors and response variables. These plots reveal the interactions among the ethanol volume fraction (A), solid-to-liquid ratio (B), ultrasound power (C), and extraction time (D). Furthermore, a consistent trend was observed for the composite indicator CE across most conditions, where it increased with all the variables before decreasing after reaching a certain threshold. Many of the 3D plots displayed a bell-shaped curve (Figure 3), with the corresponding 2D contour plots exhibiting elliptical patterns. For instance, under conditions with an ethanol volume fraction of 65% (A = 65%) and a solid-to-liquid ratio of 1:20 (B = 1:20 g/mL), the CE value peaked at 23.3503 before declining significantly. Similar trends in CE values were apparent under the other conditions.

### 2.4. DNN Results

**Figure 5:**
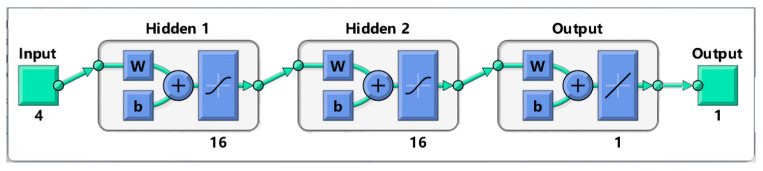
DNN model topology.

Neural networks have emerged as potent tools for simulating and optimizing the extraction processes. DNNs are founded on nonlinear mappings between dependent and independent variables and do not require prior knowledge to grasp the correlations between target responses [36]. In this investigation, the extraction time, ethanol volume fraction, solid-to-liquid ratio, and extraction power were designated as the four input layer neurons of the network, with the CE value serving as the output layer nodes. Typically, a limited number of neurons in the hidden layer can constrain the capacity of a neural network to effectively model the extraction process. Therefore, through minimization of an objective function employed for training the feed-forward network, the optimal number of hidden layer neurons was set to 16 in this study. Consequently, a four-layer (4-16-16-1) DNN model was devised, as illustrated in Figure 5.

**Figure 6:**
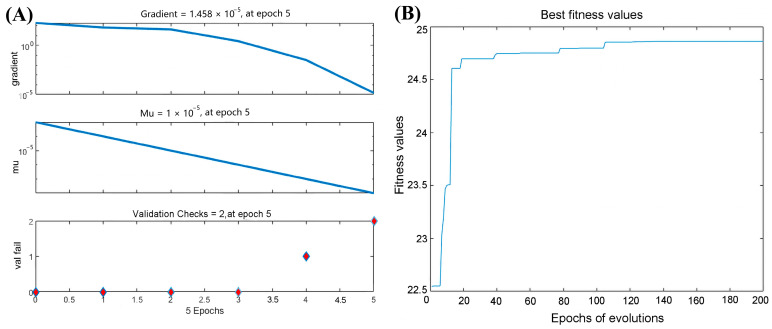
Training the DNN model using the LM algorithm (**A**) and the GA algorithm (**B**).

Randomly selected training data were employed for the DNN model, and the network was trained using the Levenberg-Marquardt algorithm (LM). Employing net.trainParam.goal = 1 × 10^−7^ as a criterion for model accuracy, the remaining network training parameters were set as follows: net.trainParam.epochs = {0, 20,000}, net.trainParam.gradient = {191, 1 × 10^−7^}, and net.trainParam. Mu = {0.001, 1 × 10^10^}, where a larger Mu value signifies an improved convergence of the algorithm. The model training state diagram is presented below, with the training conclusions occurring when the mean squared error (MSE) reaches the predefined error criterion. As depicted in Figure 6A, as the number of iterations increased, the MSE consistently remained below the specified value of 1 × 10^−7^ or lower (9.11 × 10^−14^) at net.trainParam.epochs = 5, indicating the termination of the training process with high prediction accuracy. Concurrently, the other network training parameters were set to net.trainParam.mu = 1 × 10^−8^ and net.trainParam.gradient = 1.46 × 10^−5^, with net.trainParam.validation checks = 2.

**Figure 7:**
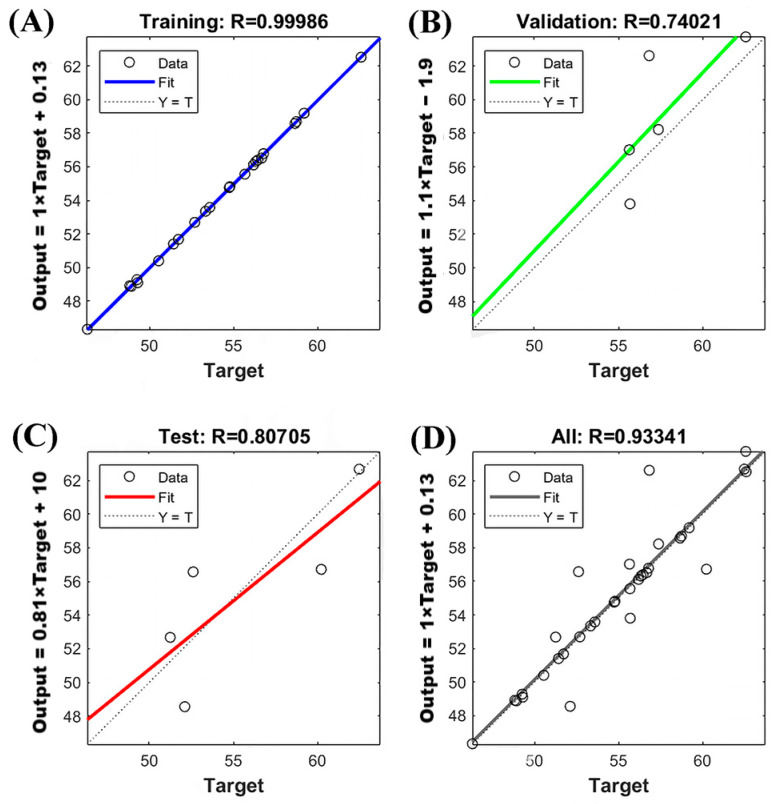
Correlation coefficient index for model construction of the DNN; Fitting equation for training set (**A**); Fitting equation for validation set (**B**); Fitting equation for testing set (**C**); Fitting equation for all sets (**D**).

With the promising performance of the neural network model established as per the preceding results, we conducted a regression analysis for both the sample and fitted values within the training and test datasets, yielding fitting curves (Figure 7). The correlation between the sample and the fitted values was most pronounced when the R^2^ of the fitted curve approached 1. By applying the model developed in this study, the following fitting equations were derived:

1. Output = 1 × Target + 0.13 (*r* = 0.99986) for the training set (Figure 7A).
2. Output = 1.1 × Target − 1.9 (*r* = 0.74021) for the validation set (Figure 7B).
3. Output = 0.81 × Target + 10 (*r* = 0.80705) for the testing set (Figure 7C).
4. Fitting equation for all sets: Output = 1 × Target + 0.13 (*r* = 0.93341) (Figure 7D).

These results underscore the efficacy of the predictive model in fitting the experimental data. The model is highly successful, with an overall R^2^ of 0.93341, indicating its robustness. Consequently, the DNN model can be relied upon for dependable predictions of the yield in ethanolic AMF extracts, particularly when employing the ultrasonic cell crusher extraction method.

Finally, the optimal parameter combination is determined using a genetic algorithm (GA), as illustrated in Figure 6B. After 100 generations, the model identifies the best fitness value. Subsequently, the fitness curve stabilizes, indicating that the optimal fitness value for the DNN model is 24.714, which remains relatively constant.

### 2.5. Validation of RSM and DNN Models

To validate the reliability of the proposed model, three experiments were conducted to determine the optimal conditions predicted by both the RSM and DNN approaches.

For RSM, the optimal process conditions calculated using Design Expert 12.0, were as follows: ethanol volume fraction: 71.787%, solid-to-liquid ratio: 1:22.115 (g/mL), ultrasonic power: 9.312%, extraction time: 36.044 min. Under these conditions, the theoretical values of TFC, rutin, Hyp, Iso, and Que were 62.315, 1.301, 11.598, 6.231, and 0.765 mg/g, respectively, whereas the theoretical value of CE was 23.218. After adjusting the extraction process in conjunction with the experimental conditions, the parameters became: ethanol volume fraction: 71%, solid-to-liquid ratio: 1:22 (g/mL), ultrasonic power: 9%, extraction time: 36 min. Following three parallel experimental validations, the results are presented in Table 4. The CE value obtained from the actual tests was 22.98 (RSD% < 1%).

For the DNN, the extraction process was globally optimized, resulting in the following optimal extraction conditions: ethanol volume fraction: 66.32%, solid-to-liquid ratio: 1:20.64 (g/mL), ultrasonic power: 9.42%, extraction time: 35.39 min. The theoretical value of CE was 24.86. Taking practical factors into account, the modified extraction conditions were adjusted to: ethanol volume fraction: 66%, solid-to-liquid ratio: 1:21 (g/mL), ultrasonic power: 9%, extraction time: 35 min. Three parallel experiments were conducted to verify these conditions, and the results are documented in Table 4, revealing that the actual CE value obtained from the experiments was 23.14 (RSD < 1%).

Table 4 illustrates that the standard deviations between the validation experimental values and the predicted values from both the RSM and DNN models were minimal. This indicates that the accuracy of both the models is relatively high. Therefore, it is reasonable and feasible to employ the RSM and DNN models for the prediction and optimization of flavonoid constituent extraction from AMF.

### 2.6. Comparison of the Developed RSM and DNN Models

Upon comparing the statistical parameters, notably the coefficient of determination (R^2^), it became evident that the DNN model outperformed the RSM model, as it exhibited a higher R^2^ value (0.9334 > 0.9241). This higher R^2^ value suggests that the DNN model is more accurate and better suited for predicting experimental conditions involving multiple dependent variables. This advantage stems from the ability of the DNN model to approximate nonlinear systems, whereas the RSM model is limited to binomial fit regression.

The performance of a model is influenced by various factors, including the modeling process and number of experiments conducted. The DNN model outperformed the RSM model in predicting experimental conditions with multiple independent variables. By comparing the experimental and predicted values of the RSM and DNN models, it is evident that the DNN model can predict such conditions. As a result, it could effectively and reliably predict and optimize the extraction process of flavonoid components in AMF, regardless of the size of the experimental dataset. Conversely, the RSM model, which is relatively simple to construct and requires only a straightforward analytical step, is more suitable for scenarios in which the number of experiments is limited. In contrast, the DNN model demonstrates versatility and performs well with both smaller and larger amounts of experimental data because it can readily accommodate the addition of new experimental data during model generation.

### 2.7. Determination Results of Flavonoids 

The regression equations, correlation coefficients, and methodological examination data for TFC, rutin, Hyp, Iso, and Que are shown in Table 5.

**Figure 8:**
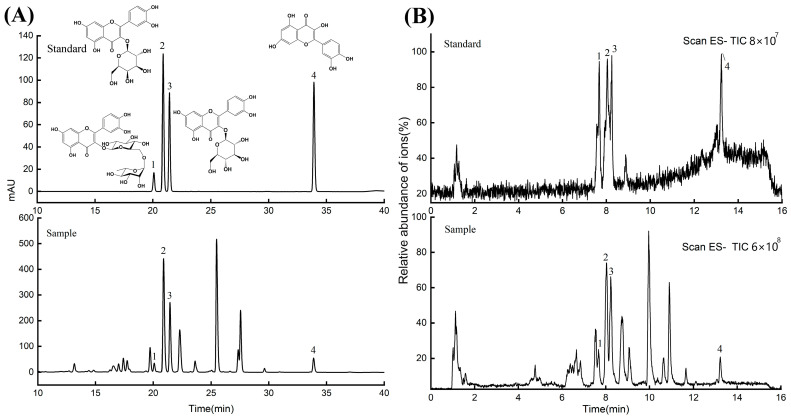
HPLC (**A**) and TIC (**B**) plots of AMF.

Figure 8 shows the HPLC chromatogram and UPLC-MS total ion flow diagram of the AMF extracts. Notably, the retention times of the individual monomeric components in the HPLC chromatograms of the sample closely matched those of the standard product. Similarly, the total ion flow diagrams of the UPLC-MS analysis of the four monomeric components also aligned with the standards. The initial analysis confirmed the presence of these four monomer components in the sample.

**Figure 9:**
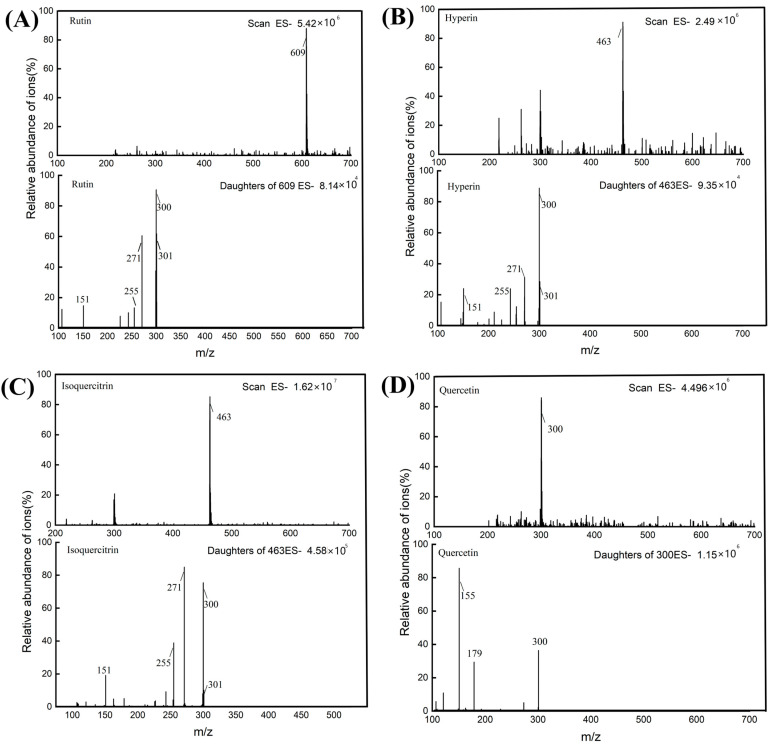
Scan ES- and daughters of ES- of four flavonoids. (**A**) Rutin, (**B**) Hyp, (**C**) Iso, (**D**) Que.

Subsequently, using precise molecular weight data obtained via UPLC-MS, along with deduction of possible elemental compositions and insights into the types and relative abundances of cleaved fragments through multistage mass spectrometry, the flavonoid compounds in the AMF extracts were identified with a high degree of confidence. The structures of the identified flavonoids are presented in Figure 9 and the ESI mass spectral parameters of the four flavonoids isolated from AMF are listed in Table 6.

Compound **1**, with a parent ion at *m*/*z* 609, was deduced to possess an elemental composition of C_27_H_30_O_16_ and was identified as a flavonol glycoside. A common characteristic of flavonol glycosides is the initial loss of sugar groups during cleavage [37]. In the ESI-MS^n^ spectrum, the parent molecular ion cleavage resulted in ions at *m*/*z* 300 and m/z 301, with subsequent retro-Diels–Alder (RDA) cleavage generating an ion at *m*/*z* 151. These mass spectral data correspond to rutin, as supported by a comparison with the mass spectral information in the literature [38]. Figure 9A displays the EI-MS^n^ mass spectrum of rutin.

Compounds **2** and **3**, with parent ions at *m*/*z* 463, were determined to have an elemental composition of C_21_H_20_O_12_ and were identified as flavonol glycosides. Similarly, these compounds exhibited a characteristic loss of sugar groups during cleavage [37]. In the ESI-MS^n^ spectrum, quasi-molecular ion cleavage yielded ions at *m*/*z* 301 and 300, followed by the loss of a molecule of CO through cleavage, resulting in an ion at *m*/*z* 271. Further cleavage led to the loss of a H_2_O molecule, producing an ion at *m*/*z* 255. The RDA cleavage generated *m*/*z* 151 ions. These mass spectral data correspond to Hyp and Iso, respectively, as confirmed by comparison with mass spectral information in the literature [38]. The ESI-MS^n^ mass spectra of Hyp and Iso are shown in Figure 9B and Figure 9C, respectively.

Compound **4**, featuring a parent molecular ion [M-H]^−^ at *m*/*z* 300, was deduced to have an elemental composition of C_15_H_10_O_7_ and was identified as a flavonol glycoside. In accordance with the literature, RDA cleavage of flavonol glycosides typically produces ions at *m*/*z* 151 and 179 [37]. Comparison with the literature mass spectral information verified compound 4 as a Que [38]. Figure 9D shows the ESI-MS^n^ mass spectrum of Que.

### 2.8. Inhibition of S. mutans Activity

**Figure 10:**
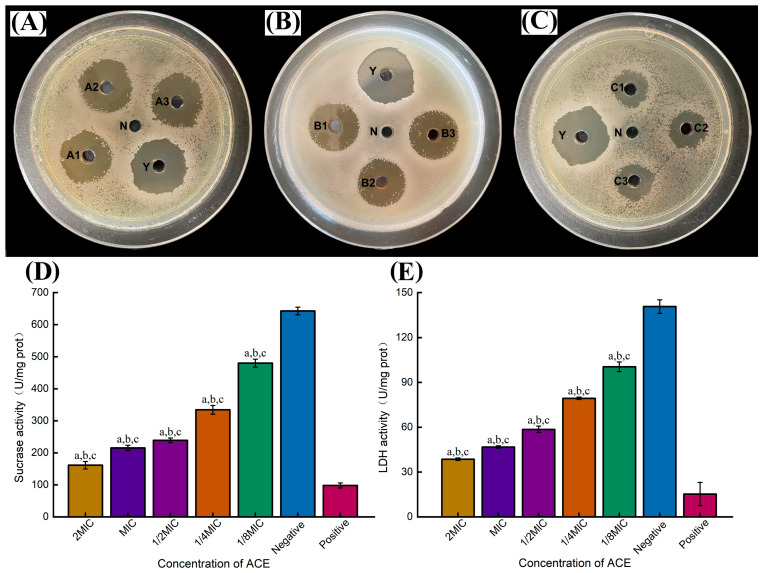
(**A**–**C**) Inhibition zone against *S. mutans*. (Note: N represents negative control, Y represents positive control, A1–A3, B1–B3, and C1–C3 represent 50, 25, and 12.5 mg/mL AMFE solution, respectively). (**D**) Inhibitory effect on sucrase. (**E**) Inhibitory effect on LDH. (Note: a, b represents a statistically significant difference compared to a negative control and a positive control at *p* < 0.001 respectively, c indicates a statistically significant difference between any two experimental groups at *p* < 0.001).

The sensitivity of the antimicrobial substance was determined by measuring the diameter of the inhibition circle during the drug sensitivity test. A diameter exceeding 20 mm indicates high sensitivity, 10–20 mm reflects medium sensitivity, and less than 10 mm indicates insensitivity. At an AMFE concentration of 50 mg/mL, the inhibition circle diameter against *S. mutans* exceeded 20 mm (22.30 ± 0.06 **), signifying high sensitivity and approaching that of the chlorhexidine positive control group (23.10 ± 0.08) (Figure 10A). Concentrations of 25 and 12.5 mg/mL resulted in medium sensitivity, with inhibition diameters of 17.87 ± 0.04 ** and 13.53 ± 0.06 **, respectively (Figure 10B,C). A concentration-dependent effect was evident, with the diameter of the inhibition circle gradually decreasing as AMFE concentration decreased. Here, ** indicates *p* < 0.001 compared to the positive control group. Furthermore, the MIC of AMFE against *S. mutans* in the planktonic state was 3 mg/mL, whereas the MBC was 6 mg/mL.

Figure 10D illustrates the dose-dependent inhibitory effect of various mass concentrations of AMFE on sucrase activity in *S. mutans* cells, with statistical significance (*p* < 0.001). Sucrase activity within the extracellular matrix of *S. mutans* was notably diminished in the 1/8MIC, 1/4MIC, 1/2MIC, and MIC groups compared to the negative control group (*p* < 0.001). These results suggest a considerable reduction in intracellular sucrase activity in *S. mutans* due to AMFE, with a more pronounced inhibitory impact observed at higher concentrations of the AMFE. Sucrase, an enzyme crucial for the breakdown of sucrose into glucose and fructose, serves as a vital nutrient source for cariogenic bacteria [39]. The suppression of sucrase activity plays a pivotal role in the prevention of dental caries. This decrease in sucrase activity indicates a diminished capacity of *S. mutans* to metabolize carbohydrates into organic acids within the oral environment, thereby lowering the susceptibility to dental caries.

Figure 10E illustrates a significant reduction in LDH activity in *S. mutans* when exposed to AMFE in the planktonic state (*p* < 0.001). LDH activity decreased from 100.466 U/mg prot to 38.622 U/mg prot across the AMFE concentration range of 1/8 MIC to 2 MIC, in comparison to the negative control group (140.652 U/mg prot). Pyruvate, a pivotal component in bacterial sugar metabolism, serves as a precursor for the production of organic acids like lactic and acetic acids by cariogenic bacteria, fostering an acidic milieu conducive to the development of dental caries [25]. LDH catalyzes the conversion of pyruvate to lactate, and the inhibition of LDH activity can lead to a reduction in lactate production, thereby diminishing the risk of dental caries. The observed reduction in LDH activity in *S. mutans* following exposure to AMFE suggests the potential of AMFE to attenuate acid production by the bacteria. This, in turn, can lead to a decrease in the acidity of the oral environment, mitigating enamel demineralization and lowering the likelihood of dental caries occurrence.

Through the literature review, the antibacterial activity of AMFE against *S. mutans* can be attributed to the presence of specific monomeric components, such as the flavonoids quercetin and rutin. Previous studies have shown that these flavonoids have significant antibacterial effects against various bacteria, including *S. mutans*. Additionally, the inhibitory effects of AMFE on LDH and sucrase may also be related to the presence of quercetin and rutin [40]. It has been reported that quercetin and rutin have inhibitory effects on these enzymes, which play a crucial role in bacterial metabolism and virulence. Therefore, the antibacterial and enzyme inhibitory effects of AMFE can be attributed to the flavonoid monomeric components quercetin and rutin, highlighting their potential as natural antibacterial agents with promising therapeutic applications.

### 2.9. Antioxidant Results

**Figure 11:**
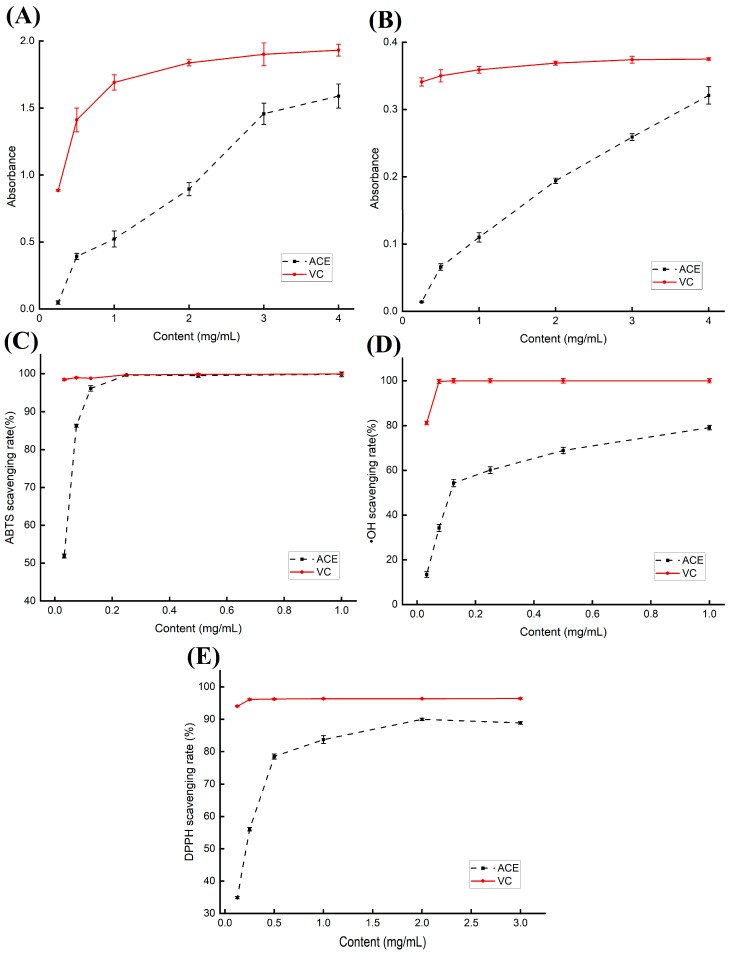
Antioxidant capacity of the AMF extracted by optimized methods; total restoration capability (**A**), total antioxidant capacity (**B**), ABTS radical scavenging capability (**C**), ·OH radical scavenging capacity (**D**), and DPPH radical scavenging capability (**E**).

In this study, the antioxidant activity of AMFE was assessed using multiple methods including potassium ferricyanide reduction, phosphomolybdenum complexation, ABTS, salicylic acid, and DPPH assays. As illustrated in Figure 11, the antioxidant capacity, as determined by these five methods, exhibited a positive correlation with increasing AMFE concentrations.

Figure 11A shows the results of the potassium ferricyanide reduction assay, which assesses AMFE’s ability of AMFE to reduce Fe^3+^. A higher absorbance indicates a stronger reduction capability. The data showed that AMFE demonstrated a significant reduction potential, with an absorbance reaching 1.50 at an AMFE concentration of 4 mg/mL.

Figure 11B presents the results of the phosphomolybdenum complexation method. This assay confirmed AMFE’s in vitro antioxidant capacity of AMFE, with absorbance gradually increasing in proportion to mass concentration.

The ABTS radical scavenging assay results, depicted in Figure 11C, revealed AMFE’s ability of AMFE to scavenge ABTS radicals. The scavenging rate increased notably with increasing mass concentration, reaching 99.19% at an AMFE concentration of 0.25 mg/mL. Within this range, the scavenging capacity of AMFEs was comparable to that of ascorbic acid (VC).

Figure 11D shows the results of the ·OH radical scavenging assay, demonstrating the ability of AMFE to neutralize ·OH radicals, with the scavenging ability progressively improving with increasing mass concentration.

Figure 11E shows the DPPH radical scavenging ability of AMFE. It effectively scavenged DPPH radicals with the highest scavenging capacity (90.12%) observed at a concentration of 2.00 mg/mL. At this concentration, the AMFE scavenging rate closely approximated that of the control VC (ascorbic acid), which was 95.56%. These findings highlight AMFE’s robust antioxidant properties of AMFE.

The robust antioxidant properties of AMFE were demonstrated through its capacity to reduce Fe^3+^ in the potassium ferricyanide reduction assay, scavenge ABTS and DPPH radicals, and neutralize ·OH radicals. These findings suggest that AMFE acts as a potent natural antioxidant, crucial for human health. Plant extracts, particularly flavonoids, have gained attention as bioantioxidants due to their numerous benefits. Flavonoids, essential secondary metabolites in plants, possess potent antioxidant properties attributed to their phenolic hydroxyl groups that interact with free radicals through hydrogen donation or acceptance, leading to the formation of stable semiquinone radicals and halting free radical chain reactions. Over time, flavonoid compounds have been recognized as crucial antioxidants, highlighting the significance of AMFE, rich in flavonoid compounds, in its antioxidant potential against oxidative stress. In conclusion, the antioxidant activity in AMFE is primarily attributed to its abundance of flavonoid compounds. Numerous studies have indicated the presence of various flavonoid components in AMF, such as quercetin, myricetin, quercetin-3-rutinoside, quercetin-3-glucoside, and kaempferol-3-rutinoside [41]. These flavonoid compounds exhibit significant antioxidant activity, effectively scavenging DPPH free radicals, ABTS free radicals, superoxide anions, and possessing reducing capabilities to protect cells from oxidative damage [42]. Overall, flavonoid compounds in AMF exert antioxidant effects through multiple mechanisms. For instance, quercetin and other flavonoid compounds can directly react with free radicals, thereby preventing damage to cells [43]. Thus, the antioxidant activity in AMFE primarily originates from its abundant flavonoid compounds, which function by directly eliminating free radicals and other means.

## 3. Materials and Methods

### 3.1. Chemicals and Reagents

Rutin (batch NO. 100050-200707), hyperin (batch NO. HR18604B1), isoquercitrin (batch NO. CFAE-536954-5G), and quercetin (batch NO. 100081-200907) were obtained from Sigma-Aldrich Company (St. Louis, MO, USA).

Methanol, acetonitrile, and formic acid (chromatographic pure) were purchased from Herbest Bio-Tech Company (Baoji, China).

LDH kit and sucrase kit were obtained from Solarbio Science & Technology Co., Ltd. (Beijing, China).

Other reagents of analytical purity are purchased from Fuyu Chemical Co., Ltd. (Tianjin, China).

### 3.2. Raw Material Preparation

The AMF used in this experiment was collected from Bozhou, Anhui Province, China. These flowers were identified by Prof. Xu Haiyan, who served as the director of the Department of Chinese Medicine Identification within the School of Traditional Chinese Medicine at Xinjiang Medical University, Urumqi, China.

In the experimental process, the flowers were crushed and subsequently passed through an 80-mesh sieve, resulting in particle sizes smaller than 180 μm. The powdered samples were dried in a desiccator. The strain of *Streptococcus mutans* (ATCC 700610) employed in this study was sourced from the Cooperative Innovation Center of Xinjiang Medical University, Urumqi, China.

### 3.3. Extraction Procedure

The extraction procedure was conducted using an ultrasonic cell crusher (JY99-IIDN; Xinzhi Biotechnology Co., Ltd., Ningbo, China). Approximately 1 g of powdered AMF was introduced into a 50 mL centrifuge tube, and ethanol was added in accordance with the designated solid-to-liquid ratio. Extraction was performed with a specified ultrasonic power and duration. Following extraction, centrifugation and filtration steps were performed, resulting in the collection of the supernatant for subsequent procedures.

### 3.4. Comparison with Two Traditional Extraction Methods

Maceration and heated reflux are two conventional methods that are frequently employed for the extraction of active constituents. Within this experimental framework, we conducted a comparative evaluation of the impact of the ultrasonic cell crusher extraction method in contrast to the two conventional extraction techniques concerning the content of key constituents, specifically TFC, rutin, Hyp, Iso, and Que, as well as the structural integrity of the AMF.

The ultrasonic cell crusher method entailed precise measurement of 1 g of AMF powder, which was subsequently mixed with 22 mL of 66% ethanol. The resulting mixture was introduced into a conical flask and subjected to a probe-type ultrasonic cell crusher for 35 min. The operational parameters were configured at a power level of 9%.

For the maceration method, 1 g of AMF powder was accurately weighed. This was combined with 30 mL of 60% ethanol, and the composite was left undisturbed at room temperature for 120 min with intermittent agitation every 5 min [44].

For the heated reflux method, 1 g of AMF powder was meticulously weighed and deposited in a 50 mL round-bottomed flask. This was combined with 50 mL of 70% ethanol and the resultant mixture was refluxed at a temperature of 90 °C in a water bath for 30 min [45].

Quantification of TFC, rutin, Hyp, Iso, and Que across various extraction methodologies was performed in accordance with the guidelines outlined in Section 3.6. Simultaneously, we scrutinized the morphological characteristics of cellular organization structures under two distinct imaging modalities: optical microscopy (OM) using an Eclipse Ni-U instrument from Nikon, Tokyo, Japan and scanning electron microscopy (SEM) employing a JSM-6390LV device (JEOL) from Akishima, Japan. Subsequently, the optimal extraction methodology was selected based on these comprehensive assessments.

### 3.5. Optimization of the Extraction Process

#### 3.5.1. Single-Factor Experiment

A one-way experiment was conducted to investigate four factors: ethanol volume fraction, solid-to-liquid ratio, ultrasonic power, and extraction time, each with five sequentially set levels. The ethanol volume fractions were 55%, 65%, 75%, 85%, and 95%. The solid-to-liquid ratios were 1:10, 1:20, 1:30, 1:40, and 1:50 g·mL^−1^. The ultrasonic power levels were set at 5%, 10%, 15%, 20%, and 25%, while the ultrasonic time levels ranged from 10 to 50 min. Three parallel trials were performed for each experiment.

The effect of each factor on the extraction was analyzed based on the experimental results. The initial range for each factor was determined by comparing the TFC, rutin, Hyp, Iso, and Que contents at different levels and calculating the comprehensive evaluation (CE) values. These findings serve as crucial references for subsequent process optimization and parameter adjustment.

#### 3.5.2. Calculation of CE Using the Entropy Weight Method

In multi-index CE, determining the weights is of utmost importance. The entropy weight method (EWM) is a CE technique that calculates the weight of each indicator by considering the information entropy between indicators [46]. Its principle is rooted in information entropy, where greater entropy indicates greater uncertainty and vice versa. The EWM is advantageous because it considers the correlation and importance between indicators, avoids subjective weighting bias, and enhances objectivity and accuracy in the analysis results.

Applying EWM to RSM and DNN accounts for the relative importance of each input variable, thereby improving the predictive capabilities. In this study, TFC, rutin, Hyp, Iso, and Que were selected as the indicators. The EWM was employed to calculate the weight coefficients (W_j_) for each component and determine the CE scores. The steps for the CE calculation are as follows:

Constructing the Evaluation Indicator System: The indicators are identified and normalized to ensure a consistent scale and value range for each indicator.

Calculating the Indicator’s Information Entropy: For each indicator, calculate its information entropy using Equation (2).

Calculate Indicator Weight: Determine the weight of each indicator based on its information entropy, as shown in Equation (3).

Comprehensive Evaluation: Multiply the weight of each indicator by its corresponding normalized value and sum to obtain the CE result.

(2) $$E_{j}=-k\sum_{j=1}^{n}p_{ij}lnP_{ij},k=1/ln\;n$$

where E_j_ represents the information entropy of the i-th indicator and P_ij_ represents the normalized value of the i-th indicator.

(3) $$W_{j}=(1-E_{j})/\sum_{i=1}^{m}\left(1-E_{j}\right)$$

where W_j_ represents the weight of the i-th indicator.

#### 3.5.3. Box–Behnken Design RSM Experiment

The Box–Behnken design (BBD) represents a response surface design methodology that employs a three-level fractional-order design and is well suited for medium-sized experiments [47]. It effectively reduces the number of required experiments while optimizing the impact of multiple input variables on the output response, thereby enhancing experimental efficiency. Notably, the BBD maintains an equidistant spacing between the levels for each variable, ensuring design equilibrium. Furthermore, the BBD employs a quadratic model to fit the response surface, enhancing the prediction of the effects of the input variables on the output response.

In the study’s response surface methodology (RSM) design, a one-way trial was structured involving four variables: A (ethanol volume fraction, %), B (solid-to-liquid ratio, g/mL), C (ultrasound time, min), and D (ultrasound power, %), each with three levels: A (55%, 65%, and 75%), B (1:10, 1:20, and 1:30 g/mL), C (10, 20, and 30 min), and D (5%, 10%, and 15%). A total of 29 experiments were conducted, comprising 5 central and 24 factorial points, as outlined in Table 2. CE serves as a comprehensive evaluation index for determining the optimal flavonoid extraction process parameters.

Mathematical modeling of the experimental data included experimental runs, regression analysis, model evaluation, optimization, and final model validation using Design Expert statistical software (version 12.0; Minneapolis, MN, USA). Furthermore, the second-order polynomial equation (Equation (4)) was employed to construct an empirical model linking the response values to the independent variables. The degree of model fit was gauged by the correlation coefficient (R^2^), and the statistical significance of the regression coefficients was assessed using the F test. Results were considered statistically significant when the *p* value was less than 0.05.

(4) $${Y=\beta }_{0}+\sum_{i=1}^{3}\beta_{i}X_{i}+\sum_{i=1}^{3}\beta_{ii}X_{i}^{2}+\sum_{i=1}^{3}\sum_{j=i+1}^{3}\beta_{ij}X_{i}X_{j}$$

where Y represents the dependent variable of the independent response (A–D), X_i_ and X_j_ represent independent variables, β_0_ represents the intercept, β_i_ is the linear coefficient, β_ii_ is the quadratic coefficient, and β_ij_ is the coefficient of the interaction term.

#### 3.5.4. Establishment of the DNN Model

The experimental dataset employed for constructing the RSM underwent DNN modeling using MATLAB software (R2020b). To mitigate the risks of overtraining and overparameterization, the total dataset comprising 25 sets was partitioned randomly into training (70%), validation (15%), and testing (15%) sets. Prior to calculations, the input data were normalized to enhance neural network performance.

The selection of an appropriate neural network topology is a pivotal aspect of successful neural network applications. The number of neurons in the input and output layers was determined based on the experimental design, and determining a suitable number of hidden layers and neurons within them requires trial and error. DNNs can encompass multiple hidden layers without constraining the node count of the hidden layer, and a two-layer DNN can accommodate arbitrary decision boundaries with proper activation functions, fitting arbitrary nonlinear mappings accurately. Consequently, the initial configuration of the number of hidden layers in this study was set to two.

Based on the aforementioned considerations, the neural network topology is specified as 4-h1-h2-1:

1. The input layer represents the data input mode with four neurons corresponding to the four input variables.
2. The number of neurons in the two hidden layers are denoted as h1 and h2.
3. The third layer constitutes the output layer, which is responsible for generating the predicted value of the target response.

The Tanh function (Equation (5)), which is characterized by its nonlinearity and S-shaped curve, serves as the chosen activation function for the DNN. It offers the advantage of output values ranging from −1 to 1, making it suitable for handling data with positive and negative values. Its nonlinear characteristics enable it to address complex nonlinear relationships, thereby augmenting a network’s expressive capabilities. In addition, the tanh function exhibits faster output changes when the input approaches zero, providing more sensitive feedback.

The Levenberg–Marquardt (LM) algorithm, a hybrid of the gradient descent and Gauss–Newton methods for forward neural network training, was selected. This algorithm combines the global convergence properties of gradient descent with the rapid local convergence of Gauss–Newton. The “trainlm” function, based on the LM algorithm, eliminates the need for Hessian matrix computations, achieving the fastest convergence rate, high accuracy, and reduced mean square error compared to other algorithms. Thus, “trainlm” is adopted as the training function for the DNN training process. Finally, the optimal individual fitness value is sought through a genetic algorithm (GA), achieved by iteratively performing selection, crossover, mutation, and evaluation steps until a stopping condition is met (e.g., reaching the maximum number of iterations). This process enables the genetic algorithm to assist in identifying the optimal hyperparameter combination on top of an existing deep neural network model, thereby enhancing the model’s performance and generalization ability.

(5) $$tanh(x)=\frac{\left(e^{x}-e^{-x}\right)}{e^{x}+e^{-x}}$$

where e is the base of natural logarithms.

### 3.6. Determination of Flavonoid Compounds

#### 3.6.1. TFC

The TFC was measured using the absorbance method described in the literature [48]. First, an accurate amount of AMFE (1 mL) was placed in a 25 mL volumetric flask, and then 50% methanol was added to bring the volume to the scale, mixed thoroughly, and left to stand for 15 min. A blank solution was prepared with 50% methanol. The absorbance value was measured at 360 nm, and a standard curve was generated using the concentration as the horizontal coordinate and the absorbance value as the vertical coordinate, with Hyp as the control.

#### 3.6.2. Determination of Rutin, Hyp, Iso, and Que by HPLC

High-performance liquid chromatography (HPLC) was used to determine the content of rutin, Hyp, Iso, and Que in AMF. First, 5 mL of AMFE supernatant was filtered through a 0.45 μm polytetrafluoroethylene (PTFE) membrane filter and then analyzed and determined using an HPLC system (1200, Agilent Technologies, Waldbronn, Germany). A Shimadzu C18 column (250 mm × 4.6 mm, 5 μm) was used as the chromatographic column. The mobile phases were 0.5% (*v*/*v*) phosphoric acid (solvent A) and acetonitrile (solvent B), at a flow rate of 1.0 mL/min. Repeated modifications were made according to the following gradient elution conditions: 0–2 min, 10% B; 2–18 min, 10% B to 20% B; 18–30 min, 20% B to 30% B; 30–35 min, 30% B; 35–35.5 min, 30% B to 10% B; 35.5–45.5 min, 10% B. The detection wavelength was 360 nm on a photodiode array detector and the column temperature was set at 30 °C with a sample volume of 10 μL.

#### 3.6.3. Characterization of Rutin, Hyp, Iso, and Que by UPLC-MS

Four flavonoids, rutin, Hyp, Iso, and Que, were analyzed and identified in the samples using ultra-performance liquid chromatography–mass spectrometry (UPLC-MS).

UPLC chromatographic conditions: An ACQUITY UPLC BEH C18 column (Waters, Milford, MA, USA, 2.1 mm × 100 mm, 1.7 µm, 130 Å) was used for analysis. The mobile phases were a 0.1% (*v*/*v*) formic acid solution (solvent A) and acetonitrile (solvent B). The optimized gradient elution program was as follows: 0–0.6 min, 10% B; 0.6–7 min, 10–20% B; 7–11.8 min, 20–30% B; 11.8–13.8 min, 30% B; 13.8–14 min, 30–10% B; and 14–16 min, 10% B. The flow rate was 0.20 mL/min. The detection wavelength was 360 nm. The injection volume was 5 μL and the column temperature was maintained at room temperature.

MS conditions: An electrospray ionization source was used for negative ion mode detection. *m*/*z*: 100–700 for MS scans and sub-scans. The capillary voltage was set to 3 kV. The temperature of the source was maintained at 150 °C. The dissolution temperature was set at 350 °C. A Waters TQ Detector was used for analysis.

### 3.7. Inhibitory Activity of AMFE against S. mutans

The *S. mutans* strain was removed from the refrigerator at −80 °C, diluted with BHI liquid medium, and resuspended under anaerobic conditions at 5% CO_2_ and 37 °C for 48 h. The resuscitated strain was inoculated into BHI solid Petri dishes for incubation using the plate scribing method and colony counting was performed. Finally, the bacterial concentration was adjusted to 10^8^ CFU/mL with the BHI culture solution, and the bacterial concentration was adjusted as needed.

#### 3.7.1. Inhibition Circle Test

The agar perforation method was used to determine the diameter of the AMFE inhibition circle at different concentrations [49]. First, 100 μL of BHI bacterial suspension at a concentration of 10^6^ CFU/mL was aspirated using a pipette gun and coated evenly on the BHI solid medium. After the coated bacterial suspension was dried, five holes with a diameter of 6 mm were punched into the BHI solid medium using a hole punch. Next, 40 μL of the sample solution/complex chlorhexidine solution (positive control)/sterile water (negative control) was added to each well. Next, the culture was incubated under anaerobic conditions at 5% CO_2_ and 37 °C for 24 h. Finally, the diameter of the circle of inhibition was measured using a millimeter Vernier caliper and the experiment was repeated three times.

#### 3.7.2. Determination of Minimum Inhibitory Concentration (MIC) and Minimum Bactericidal Concentration (MBC)

The MIC and MBC of AMFE against *S. mutans* were determined by the microdilution method [50]. First, a 50 mg/mL solution of AMFE was prepared, which was aseptically filtered and then diluted to a concentration of 50–1 mg/mL using sterile ultrapure water. Then, 100 μL of bacterial suspension (10^7^ CFU/mL) with 100 μL of AMFE solutions of different concentrations was added to 96-well plates and mixed well. The plates were then incubated under anaerobic conditions at 5% CO_2_ and 37 °C for 24 h. A blank control group of bacterial suspension plus sterile water was set up simultaneously, and three parallel groups were established for each group. Twenty-four hours later, the plates were removed and uncovered for observation, and the minimum concentration of the extract with no *S. mutans* growth in the wells was recorded as the MIC. After the MIC was determined, bacterial suspensions with a concentration ≥MIC in 96-well plates were selected, and the appropriate amount of the bacterial suspension was pipetted with a sterile pipetting gun. The bacterial suspension was inoculated on a new BHI solid medium (5% CO_2_) and incubated anaerobically at 37 °C for 24 h. MBC was recorded as the extract concentration with no *S. mutans* growth on the BHI solid medium.

#### 3.7.3. The Inhibitory Effect of AMFE on Sucrase in *S. mutans*

In this study, 3,5-dinitrosalicylic acid was used to determine the inhibitory effect of AMFE on sucrase activity in *S. mutans* [51]. This method is based on the principle that 3,5-dinitrosalicylic acid is reduced to a brownish-red amino compound together with a reducing sugar, and the amount of reducing sugar is proportional to the depth of color of the reaction solution within a certain range.

The AMFE mother liquor was adjusted to different concentration solutions (2 MIC, MIC, 1/2 MIC, 1/4 MIC, and 1/8 MIC) with BHI culture solution. The bacterial suspension (approximately 1–5 × 10^6^ CFU/mL) was mixed with different concentrations of AMFE solution at 1:10 (*v*/*v*) and then removed after resuscitation for 24 h at 5% CO_2_ and 37 °C under anaerobic conditions. The mixture was centrifuged for 5 min, the bacterial precipitate was collected, and the bacterial cells were gently washed twice with PBS buffer (0.1 mol/L, pH = 6.2). Bacteria were added to 1 mL of extract from the sucrase kit (BC0135; Solarbio Science & Technology Co., Ltd., Beijing, China) according to the bacteria or number of bacteria (1–5 × 10^6^: approximately 5 million bacteria to 1 mL of extract), and the bacteria were crushed using an ultrasonic cell crusher (ice bath, 20% power, ultrasound for 3 s at 10 s intervals, repeated 30 times). Centrifugation was performed at 8000× *g* for 10 min at 4 °C, and the supernatant was collected and placed on ice for measurement. The effect of AMFE on sucrase activity in *S. mutans* was determined using a sucrase test kit, with the negative control group in a medium without AMFE and a positive control group in a medium containing 0.12% chlorhexidine. Three parallel tubes were set up for each group, and the experiment was repeated three times. Sucrase activity was defined as the catalytic hydrolysis of 1 μg of sucrose per mg of histone per minute, and the formula (Equation (6)) is shown below.

(6) $$Sucrase\;activity(U/mg\;prot)=\frac{1000\times X\times V}{V\times Cpr}÷T=100\times X÷Cpr$$

where 1000 represents 1 mg/mL = 1000 μg/mL, V represents the sample volume added to the reaction system (30 μL), T is the reaction time (10 min), and Cpr represents the protein concentration (mg/mL, determined according to BCA kit (PC0020; Solarbio, Beijing, China) instructions.

#### 3.7.4. The Inhibitory Effect of AMFE on LDH in *S. mutans*

The inhibitory effect of AMFE on lactate dehydrogenase (LDH) activity in *S. mutans* was determined by measuring the production of pyruvate catalyzed by LDH using an LDH assay kit (BC0685; Solarbio, Beijing, China) [52]. The principle of the assay is that LDH catalyzes the oxidation of NAD+ by lactate to generate pyruvate, which further reacts with 2,4-dinitrophenylhydrazine to form pyruvate dinitrophenylhydrazone, exhibiting a brownish-red color in an alkaline solution. The color intensity was proportional to the pyruvate concentration.

After inoculation, as described in Section 3.7.3, bacterial cells from different treatment groups were collected by centrifugation for 5 min, and the supernatant was collected. The effect of AMFE on LDH activity of *S. mutans* was determined using an LDH assay kit (BC0685; Solarbio, Beijing, China). LDH activity was defined as the production of 1 nmol of pyruvate per minute per mg of tissue protein. The calculation formula is given by Equation (7).

(7) $$LDH(U/mg\;prot)=\frac{y\times V}{V\times Cpr}÷T\times 1000=66.7\times y÷Cpr$$

where V represents the volume of the sample added to the reaction system (50 μL), T is the reaction time (15 min), Cpr is the protein concentration (mg/mL, determined according to the instructions in the BCA instructions (PC0020; Solarbio, Beijing, China)), and 10^3^ is the unit conversion coefficient (1 μmol/mL = 10^3^ nmol/mL).

### 3.8. In Vitro Antioxidant Activity of AMFE

The total reduction capacity(TRC) of AMFE was determined using a Fe^3+^ reducing power assay [53]. One milliliter of phosphate buffer (pH = 6.6), AMFE solution, and 1% potassium ferricyanide solution were added to the test tube at a ratio of 1:1:1 by volume, mixed homogeneously, and then reacted at 50 °C for 20 min in a water bath and cooled down. Next, 2 mL of 10% trichloroacetic acid solution was added and centrifuged at 3000 rpm for 10 min. Then, 50 μL of the supernatant, 150 μL of distilled water, and 20 μL of 0.1% ferric chloride solution were mixed in the dark for 30 min. Fifty microliters of the supernatant, 150 μL of distilled water, and 20 μL of 0.1% ferric chloride solution were mixed and placed in the dark for 30 min, and the absorbance was measured at 700 nm at the end of the reaction.

The total antioxidant activity (TAC) of AMFE was determined by the phosphomolybdenum complexation method [54]. Five hundred microliters of AMF solution with different concentrations and 1 mL of H_2_SO_4_, Na_3_PO_4_, and ammonium molybdate solutions were mixed homogeneously. The solution was then placed at a constant temperature of 95 °C in a water bath for 90 min and the absorbance was measured at 695 nm.

The ABTS radical scavenging ability of AMFE was determined using an ABTS assay with some modifications [55]. ABTS+ mother liquor was prepared, refrigerated at 4 °C for 14–16 h, and set aside. Before use, the solution was diluted with anhydrous ethanol until its absorbance was in the range of 0.7 ± 0.02 Abs. Fifty microliters of AMF solutions of different concentrations and 150 μL ABTS were added to a 96-well plate and mixed well. After 6 min of reaction at room temperature, absorbance was measured at 734 nm. The clearance was calculated using Equation (8).

The ·OH radical scavenging ability of AMFE was determined using the salicylic acid method [56]. Each sample solution (50 μL), 5 mmol/L FeSO_4_ solution, and salicylic acid-ethanol solution were added to a 96-well plate and mixed thoroughly. The reaction was carried out at room temperature for 10 min, 50 μL of a 5 mmol/L H_2_O_2_ solution was added, the mixture was left at room temperature for 30 min, and the absorbance was measured at 510 nm after the reaction was complete. The clearance was calculated using Equation (8).

DPPH assay was used to determine the DPPH radical scavenging ability of AMFE [56]. DPPH solution was prepared at a concentration of 50 mg/mL, and 50 μL of AMFE solution and 150 μL of DPPH solution were added to a 96-well plate and mixed at room temperature. The absorbance of the mixture was measured at 520 nm after 0.5 h of reaction protected from light. The clearance was calculated using Equation (8).

(8) $$ABTS(\cdot OH/DPPH)radical\;scavenging\;rate(\%)=\frac{1-(A_{2}-A_{1})}{A_{0}}\times 100\%$$

where A_2_, A_1_, and A_0_ represent the experimental, control, and standard groups, respectively.

### 3.9. Statistics

The results of the experiments were statistically analyzed using the statistical programs Design Expert 12.0, MATLAB (version R2020b), and SPSS 27.0. All data presented represent the mean ± standard deviation (SD) of at least three independent experiments (*n* = 3). The analysis of variance (ANOVA) was utilized to examine variances in the outcomes of the samples, and discrepancies were deemed significant when *p* < 0.05. The experimental and optimization statistics for the RSM were designed using Design Expert 12.0, and regression equations for evaluating the response, contribution, and significance of each parameter, response surface plots, and optimal conditions for the extraction were obtained. A DNN was constructed, tested, and validated using MATLAB. EWM was used to calculate the entropy weights of each input variable to improve the predictive ability of the two extraction processes by synthesizing the evaluation results.

## 4. Conclusions

In this study, we compared the effects of ultrasonic cell crusher, traditional ambient maceration, and heat reflux methods for isolating flavonoid constituents from AMF. Further optimization of the ultrasonic process was achieved using the RSM and DNN. Following DNN-based optimization, the optimal conditions involved 66% ethanol, a solid-liquid ratio of 1:21, 9% ultrasonic power, and 35 min extraction time. Under these conditions, the flavonoid extraction efficiency reached 23.14, with an RSD of less than 1%, exceeding the efficiencies predicted by the RSM model. In addition, preliminary in vitro data showed potent inhibition against *S. mutans* by disrupting key enzymes and demonstrated strong antioxidant properties of the resulting AMFE. These findings serve as a substantive basis for future AMF investigations.

## Figures and Tables

**Table 1:** Comparing several extraction techniques using ethanol as the solvent.

Method	Concentration of Ethanol (%)	Temp (°C)	Time (min)	Yield (Mean ± SD, mg/g)					
				TFC	Rutin	Hyp	Iso	Que	CE
Maceration extraction	60	RT	120	59.65 ± 0.18	1.14 ± 0.16	10.03 ± 0.12	5.43 ± 0.08	0.62 ± 0.12	21.78 ± 0.15
Reflux extraction	70	90	30	60.02 ± 0.11	1.12 ± 0.15	10.24 ± 0.08	5.49 ± 0.11	0.66 ± 0.05	21.97 ± 0.13
Ultrasonic crusher extraction	66	RT	35	60.86 ± 0.16	1.41 ± 0.14	11.86 ± 0.09	6.60 ± 0.12	0.77 ± 0.15	22.98 ± 0.09

Note: RT represents room temperature.

**Table 2:** Comparison of the RSM and DNN models on extraction results.

Runs	Independent Variables				Dependent Variables (mg/g)							
	A	B	C	D	TFC	Rutin	Hyp	Iso	Que	CE		
					Obs	Obs	Obs	Obs	Obs	Obs	RSM Pre	DNN Pre
1	55	10	10	30	51.25	1.10	9.50	5.09	0.21	19.07	18.80	19.07
2	75	10	10	30	56.18	1.16	10.22	5.55	0.67	20.86	20.41	20.48
3	55	30	10	30	53.58	1.19	10.74	5.77	0.62	20.86	20.71	19.92
4	75	30	10	30	59.19	1.18	10.90	5.88	0.70	22.01	21.69	22.02
5	65	20	5	20	54.78	1.13	10.15	5.45	0.64	20.40	19.91	20.40
6	65	20	15	20	52.61	1.11	10.18	5.47	0.63	19.77	19.74	19.21
7	65	20	5	40	57.37	1.25	11.34	6.12	0.70	21.67	21.11	21.68
8	65	20	15	40	56.78	1.26	11.12	5.96	0.69	21.40	21.29	21.39
9	55	20	10	20	51.72	1.16	10.50	5.61	0.62	19.68	19.74	19.67
10	75	20	10	20	56.44	1.24	11.01	5.95	0.70	21.26	20.66	15.90
11	55	20	10	40	55.64	1.17	10.37	5.56	0.30	20.73	20.75	20.75
12	75	20	10	40	58.65	1.30	11.74	6.34	0.76	22.22	22.51	22.23
13	65	10	5	30	48.91	1.16	10.20	5.53	0.61	18.71	18.82	18.71
14	65	30	5	30	56.32	1.22	11.13	5.98	0.62	21.26	21.13	21.26
15	65	10	15	30	49.29	1.18	10.49	5.67	0.68	18.94	19.54	18.94
16	65	30	15	30	52.69	1.20	10.85	5.82	0.69	20.08	20.42	20.06
17	55	20	5	30	54.74	1.15	10.49	5.61	0.64	19.63	20.21	19.63
18	75	20	5	30	56.82	1.27	11.17	6.03	0.72	21.44	21.92	21.20
19	55	20	15	30	55.67	1.20	10.92	5.86	0.65	20.98	20.63	20.96
20	75	20	15	30	58.73	1.23	11.09	6.00	0.72	21.97	21.51	21.95
21	65	10	10	20	48.82	1.12	10.01	5.38	0.63	18.59	18.48	18.58
22	65	30	10	20	52.11	1.17	10.64	5.71	0.67	19.82	19.83	19.82
23	65	10	10	40	50.54	1.25	10.97	5.91	0.70	19.51	19.62	19.51
24	65	30	10	40	55.68	1.27	11.47	6.13	0.72	21.22	21.45	21.23
25	65	20	10	30	62.22	1.26	11.08	5.92	0.70	22.97	23.07	23.08
26	65	20	10	30	63.41	1.27	11.14	5.96	0.71	23.35	23.07	23.08
27	65	20	10	30	64.10	1.31	11.55	6.18	0.73	23.72	23.07	23.08
28	65	20	10	30	61.37	1.23	10.94	5.86	0.69	22.66	23.07	23.08
29	65	20	10	30	61.82	1.27	11.14	5.96	0.72	22.66	23.07	23.08

**Table 3:** ANOVA for the response surface quadratic model of CE.

Source	Sum of Squares	df	Mean Square	F Value	*p* Value	
Model	52.60	14	3.76	14.63	<0.0001	significant
A—Ethanol concentration	5.03	1	5.03	19.58	0.0006	
B—Solid-to-liquid ratio	7.62	1	7.62	29.66	<0.0001	
C—Power	0.0001	1	0.0001	0.0002	0.9880	
D—Time	5.71	1	5.71	22.24	0.0003	
AB	0.0987	1	0.0987	0.3842	0.5453	
AC	0.1706	1	0.1706	0.6642	0.4287	
AD	0.2238	1	0.2238	0.8715	0.3664	
BC	0.5004	1	0.5004	1.95	0.1845	
BD	0.0575	1	0.0575	0.2239	0.6434	
CD	0.0317	1	0.0317	0.1235	0.7305	
A^2^	4.03	1	4.03	15.70	0.0014	
B^2^	22.94	1	22.94	89.33	<0.0001	
C^2^	9.59	1	9.59	37.33	<0.0001	
D^2^	11.75	1	11.75	45.74	<0.0001	
Residual	3.60	14	0.2568			
Lack of Fit	2.75	10	0.2751	1.30	0.4299	not significant
Pure Error	0.8446	4	0.2111			
Cor Total	56.20	28				
Fit Statistics						
R^2^	0.9241					
R^2^_Adj_	0.8883					
R^2^_Pre_	0.7418					
Adeq Precision	13.1487					
C.V%	2.25					

**Table 4:** The predicted and verification results of the responses under the optimum conditions according to the analysis by RSM and DNN models. ($\overset{—}{X}$ ± SD) (*n* = 3).

Variables		RSM		DNN	
		Predicted	Experimental	Predicted	Experimental
Input	Ethanol concentration (%)	71.78	72	66.32	66
	Solid-to-liquid ratio (g/mL)	1:22.12	1:22	1:20.64	1:21
	Ultrasonic time (min)	36.04	36	35.39	35
	Ultrasonic power (%)	9.31	9	9.42	9
Output	CE value	23.12	22.98 ± 0.09	24.86	23.14 ± 0.10
SD (%)		0.17		1.21	

**Table 5:** Regression equations, correlation coefficients, and methodological considerations for TFC, rutin, Hyp, Iso, and Que.

NO.	Linear Regression	Concentration Range (μg·mL^−1^)	Correlation Coefficient	Precision (%)	Stability (%)	Accuracy (%)	Recovery Rate (%)
TFC	*y* = 29.382*x* + 0.0437	3.24–19.46	0.9991	0.86	0.31	0.53	1.13
Rutin	*y* = 3.4269*x* + 0.0794	9.80–49.00	0.9995	0.48	0.70	1.18	0.30
Hyp	*y* = 3.4269*x* + 0.0794	105.60–633.60	0.9989	0.64	1.26	6.75	3.74
Iso	*y* = 5.4421*x* + 15.062	69.40–408.00	0.9997	0.78	0.67	4.49	2.49
Que	*y* = 10.949*x* + 11.567	17.48–104.88	0.9997	0.28	0.39	1.34	8.42

**Table 6:** Mass spectrometry parameters of the four components in the AMF samples.

NO.	Retention (min)	Molecular Formula	Parent Ion (*m*/*z*)	Daughter Ion (*m*/*z*)	Collision Voltage (V)	Identification
1	7.643	C_27_H_30_O_16_	609	151, 300, 301	50 V	Rutin
2	8.045	C_27_H_30_O_16_	463	151, 255, 271, 300, 301	56 V	Hyperin
3	8.248	C_27_H_30_O_16_	463	151, 255, 271, 300, 301	45 V	Isoquercitrin
4	13.234	C_15_H_10_O_7_	300	155, 179	22 V	Quercetin

## Data Availability

All of the data are contained within the article.
